# “A part of my life”. A qualitative study about perceptions of female genital mutilation and experiences of healthcare among affected women residing in Sweden

**DOI:** 10.1186/s12905-024-03149-1

**Published:** 2024-05-22

**Authors:** Bita Eshraghi, Lena Marions, Cecilia Berger, Vanja Berggren

**Affiliations:** 1 Dept of Clinical Science and Education, Karolinska Institutet, Södersjukhuset, Stockholm, Sweden; 2 Department of Obstetrics and Gynecology, Södersjukhuset, Stockholm, Sweden; 3 Dept of Neurobiology, Caring Science and Society (NVS), Karolinska Institutet, Stockholm, Sweden

**Keywords:** Female genital mutilation, Interview study, Qualitative study, Sweden Healthcare

## Abstract

**Background:**

Female genital mutilation (FGM) is defined as all procedures involving partial or total removal of the external female genitalia, or other injuries to them for non-medical reasons. Due to migration, healthcare providers in high-income countries need to better understand the consequences of FGM. The aim of this study was to elucidate women’s experiences of FGM, with particular focus on perceived health consequences and experiences of healthcare received in Sweden.

**Methods:**

A qualitative study was performed through face-to-face, semi-structured interviews with eight women who had experienced FGM in childhood, prior to immigration to Sweden. The transcribed narratives were analyzed using content analysis.

**Results:**

Three main categories were identified : “Living with FGM”, “Living with lifelong health consequences” and “Encounters with healthcare providers”. The participants highlighted the motives behind FGM and their mothers’ ambivalence in the decision process. Although the majority of participants had undergone FGM type 3, the most severe type of FGM, the lifelong health consequences were diverse. Poor knowledge about FGM, insulting attitude, and lack of sensitive care were experienced when seeking healthcare in Sweden.

**Conclusions:**

Our findings indicate that FGM is a complex matter causing a diversity in perceived health consequences in women affected. Increased knowledge and awareness about FGM among healthcare providers in Sweden is of utmost importance. Further, this subject needs to be addressed in the healthcare encounter in a professional way.

**Supplementary Information:**

The online version contains supplementary material available at 10.1186/s12905-024-03149-1.

## Background

**Fig. 1:**
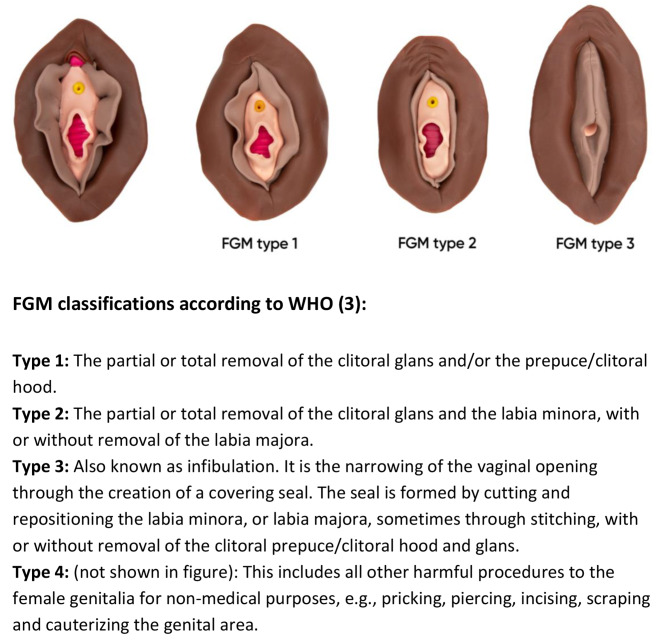
From left to right: normal anatomy of the vulva, FGM type 1, FGM type 2, FGM type 3. Clay vulva models by Josefin Herolf in collaboration with the Amel clinic*. Photo: Fotogruppen, Södersjukhuset. *Specialized FGM-clinic in Södersjukhuset, Stockholm, Sweden

The World Health Organization (WHO) defines female genital mutilation (FGM), as all procedures involving removal of or injury to the external female genitalia for non-medical reasons [1]. FGM is mostly carried out on girls between infancy and the age of 15 and the type of FGM varies between communities [1]. The four types of FGM are presented in Fig. 1. It is estimated that more than 200 million girls and women have been subjected to FGM worldwide, and that nearly four million girls are at risk annually [2]. FGM is mostly found in a cluster of countries on the African continent, with FGM prevalence as high as 98% among girls and women in Somalia. To a lesser extent, the practice is found in parts of the Middle East and Asia [2].

Complications after FGM include both immediate and long-term consequences. The immediate complications can include pain, hemorrhage, urinary problems, genital tissue swelling, infections and sometimes death [3, 4]. Long-term complications include menstrual and urinary problems, as well as sexual problems such as painful intercourse and low satisfaction [3, 5, 6]. FGM has also been associated with obstetric complications such as perineal tears, prolonged labor and episiotomy [3, 5, 7]. The experience of FGM has further been associated with adverse mental health outcomes such as depression, anxiety and post-traumatic stress disorder (PTSD) [3, 8]. It is suggested that the degree of complications is in relation to the severity of FGM [4, 8, 9].

Numerous sociocultural factors contribute to the practice and its continuation. Regardless, FGM is an expression of gender inequality rooted in social, economic and political structures (10). Where widely practiced, it is a part of the social norm and everyone is expected to comply with it. It is seen as a rite of passage that reinforces cultural identity and a sense of belonging [1]. Ensuring the girl’s chastity, marriageability and hygiene are other common motives. Although FGM is not endorsed by religion and predates Christianity and Islam, some communities consider the practice a religious requirement [1, 10, 11].

Due to migration, approximately half a million women and girls with experience of FGM now live in Europe [12]. According to the Swedish National Board of Health and Welfare’s survey (2023), it is estimated that 68,000 girls and women in Sweden live with the consequences of FGM. The largest estimated groups are born in Somalia, Eritrea, Ethiopia, Iraq, Egypt, Sudan and Gambia [13]. Although a few qualitative studies on women’s experiences of FGM and its effect on health in migrant populations have been conducted, this field is still under-researched [14–17]. FGM is a global health concern and brings healthcare challenges in countries with large FGM-affected diasporas. Healthcare services in Western countries do not seem prepared to care for girls and women affected by FGM [18, 19]. A report from the Swedish National Board of Health and Welfare describes lack of knowledge and experience of FGM among staff, language difficulties and fear of stigmatization as reasons for finding it difficult to discuss FGM with patients [20]. The report highlighted the lack of care for women with FGM and suggests further knowledge-raising initiatives among healthcare providers.

Disempowerment, poor attitude and offensive comments from healthcare providers have been described by women with FGM when seeking prenatal care in Norway and the UK [21, 22]. Studies from Sweden have reported that women with FGM sometimes feel looked down on, disrespected and not listened to by healthcare providers during pregnancy and delivery [23, 24]. They also experienced language barriers and poor knowledge about FGM among the staff. In several studies, fear of poor knowledge has been expressed by participants [22, 25, 26]. Contrastingly, women also reported positive encounters with health care providers mainly due to the midwifes’ or doctors’ good knowledge about FGM and friendly attitude [23, 25, 26].

The aim of this study was to elucidate women’s experiences of FGM, with focus on perceived consequences on their health, and experiences of encounters with healthcare providers in Sweden.

## Methods

Our method chosen was an inductive, qualitative interview study. A qualitative study design is a common choice when there is a lack of available research on an issue such as in this case [27]. A semi-structured interview method was chosen to give the participants the freedom to express their views in their own words within the topics chosen. The interviews were based on the idea that it is a dialogue between the interviewee and the interviewer [28]. The inclusion criteria for participants were age above 18, experience of FGM during childhood and Sweden as current place of residence. It was also a requirement to speak Swedish. The participants were recruited from a gynecological outpatient clinic in Stockholm, Sweden during 2019–2021. The clinic is specialized in caring for women and girls with experience of FGM. Recruitment from this specialized unit gave the opportunity to offer psychological support if needed. We recruited participants using purposeful sampling, thus asked women that we considered as emotionally robust and that could contribute with rich and in-depth information and appeared willing to share their experiences [27]. We strived for heterogeneity among the participants concerning age, ethnic group and religion. Due to inclusion criteria and purposeful sampling, not all patients consecutively visiting the clinic were eligible for participation [29]. The participants were recruited by clinicians working at the clinic. After the ordinary consultation, they were given oral and written information about the study and asked about participation, including that it was voluntary and that they could withdraw from the study at anytime. After giving written informed consent, the participant chose the location and suitable time for the interview. The interviewer was not involved in the or in care given.

The interviews were performed in parallel with the analysis of the data until saturation was achieved, i.e. when no new perspectives emerged during the analysis [30]. In the interviews conducted at the end of data collection, the stories contained variations of previously described perspectives and experiences. Since we noted that the answers did not yield any new perspectives after the sixth interview, the authors discussed the saturation and agreed to proceed with two more interviews to ensure that saturation was achieved. Due to the Covid-19 pandemic, the time between the decision to participate and the actual interview was prolonged in some cases.

Two pilot interviews were performed to ensure that the interview questions answered the aim and whether they opened the possibility for the informants to share their in-depth experiences. The pilot interviews were concluded to be rich in data and were later included in the final result. Six of the interviews took place in the participant’s home and two were conducted in a private room in the hospital where the out-patient clinic is located.

The pre-tested semi-structured interview guide also included sociodemographic data and information about FGM status (supplementary file). The topics in the interview guide where three: (1) the experience of FGM, (2) informants own thoughts about possible self-lived health complications, (3) their perceptions of the encounter with Swedish health care providers. The first author (BE) conducted all the interviews which were performed face-to-face and digitally recorded for later verbatim transcription. Each interview lasted between 45 and 90 min.

The transcribed text was analyzed using content analysis according to Graneheim and Lundman [31]. The analysis was conducted in five steps. First, all texts were read carefully, yielding an overall impression of the content. Second, in order to answer our research questions, so-called meaning units of one or more sentences within each topic were selected. Third, the underlying meanings were condensed. Fourth, the condensations were formulated into codes. The text was critically analyzed, read and compared to achieve reasonability. Lastly, the researchers reflected upon and discussed the findings, considering the research questions and agreed on the subcategories and main categories.

### Ethical approval

was obtained from the Swedish Ethical Review Authority (Dnr: 2019 − 01492). The ethical principles of the Declaration of Helsinki were followed [32]. Informed written and oral consent were obtained from the participants at recruitment, including information that they could withdraw from the study at any time.

## Results

Eight women who met the inclusion criteria agreed to take part in the study (Table 1). Their ages ranged from 25 to 36 years, and they had resided in Sweden between 10 and 30 years. A majority of participants migrated to Sweden at the age of 10 or younger. All of them spoke good or excellent Swedish. One woman originated from Eritrea, one from eastern Ethiopia (Somali region) and the remaining from Somalia. Seven of them were mothers or soon-to-be mothers. Six of the participants had a college or university degree and two had upper secondary school-level education. One participant was Orthodox Christian and the rest identified themselves as Sunni Muslims. One woman had been subjected to FGM type 2 and the remaining seven to FGM type 3. All of the infibulated women had undergone deinfibulation.

**Fig. 2:**
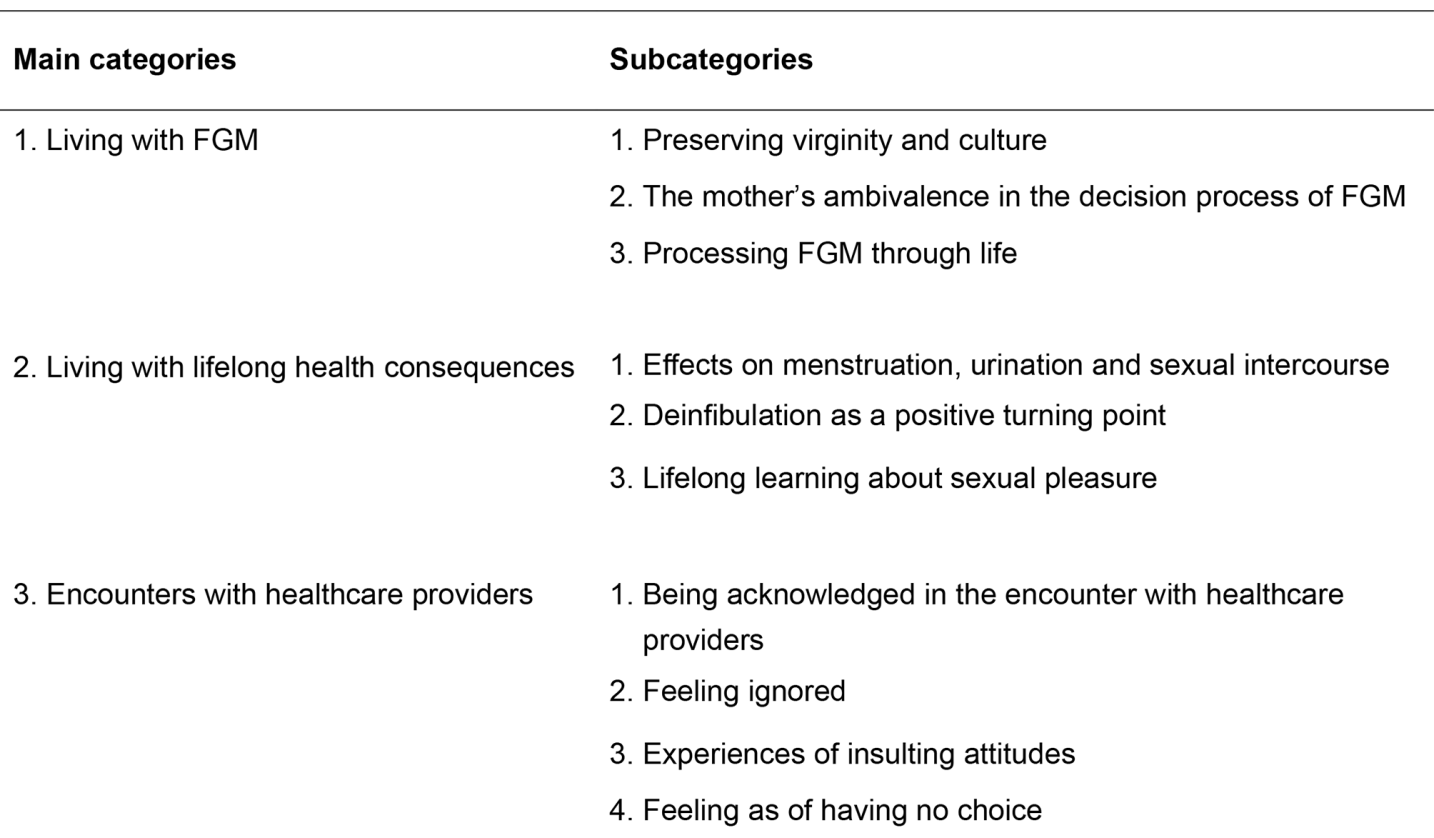
Main categories and subcategories that emerged from the analysis

During the interviews the participants were generally eloquent and eagerly shared their stories, not seldom with laughter. The names below the quotations are not the real names of the participants in this study. The three main categories and ten subcategories that emerged from the analysis are presented in Fig. 2.

**Table 1:** Characteristics of participants

Name	Age (years)	Country of origin	Length of stay in Sweden (years)	Level of education	Marital status	Religion	Age at FGM (years)
Hiba	25	Somalia	15	University	Married	Sunni Muslim	7
Senait	34	Eritrea	32	University	Partner	Orthodox Christian	< 1
Amal	36	Somalia	32	University	Singel	Sunni Muslim	6
Deeqa	29	Somalia	25	University	Married	Sunni Muslim	2
Zahra	35	Somalia	16	College	Partner	Sunni Muslim	6
Fatima	27	Somalia	10	Upper secondary school	Married	Sunni Muslim	12
Khadra	34	Somalia	30	Upper secondary school	Divorced	Sunni Muslim	2
Ayaan	34	Eastern Ethiopia, Somali region	30	University	Singel	Sunni Muslim	4

### Living with FGM

All participating women shared the experience of having undergone FGM as girls. The women’s memories of the event differed. Most participants did not know what was about to happen and were very surprised. The age at the event varied between 6 months and 12 years of age. All girls except one underwent FGM type 3. The context varied with six women being genitally mutilated in their own home or the home of a grandmother or aunt, and two of the women at health facilities. Seven women reported having a female circumciser whereas one woman reported that the procedure was performed by a male doctor. Four of the women that had the procedure performed between 4 and 12 years of age, recalled the event as something very traumatic and painful. Two of them did not receive any anesthetics. However, for those two who did and still experienced pain, this was either during the procedure and/or afterwards when passing urine. Three women had very little or recalled no memory at all from the event due to young age. One woman explained the procedure as something nontraumatic and without pain. She received anesthetics during the procedure and further was the only participant that actively had been involved in the decision of going through FGM.

### Preserving virginity and culture

The women expressed that culture was the main reason for continuation of the tradition of FGM and that the tradition is based on safeguarding and ensuring virginity. A general motivation for the tradition, which the participants had been explained to by others, was that it transforms girls into “real” women and that it was done to avoid condemnation, harassment, and ostracism.

*“It is a tradition. That women should not feel sexual desire and practice sex without being married…They believe they remove the desire to have sex, but that is not the case.”* (Ayaan).

Other arguments recalled for performing FGM were due to the force of social norms, that the girls and their families would become a part of the community, or to hurry up and ensure that it was performed before migration.

*“I personally remember wanting to go through it because everyone my age had it done. It was such a thing that if you didn’t have it done, you were ashamed. You wanted to feel like a woman, you wanted to be a part of the gang… I wanted to do it, it was really a decision I took… Also, I was influenced by the opinions of others, influenced by society’s opinions and the culture. You want to be like your mother, your sister, your grandmother.”* (Hiba).

The participating women mentioned that there is no clear religious argument for FGM, except one woman who mentioned pricking from religious Islamic texts (Hadiz). Although culture and tradition emerged as a central motive from the interviews, this was also questioned within the family. It was described that the tradition was continued, despite the knowledge of the harm it resulted in, because of the perceived social benefits.

*“This is the way it always has been. The tradition just continues, without being questioned. It was never because of Islam…God created you just like you are, you don’t need to change anything. So, I think if you follow that literally, this would never exist.”* (Hiba).

### The mother’s ambivalence in the decision process of FGM

The motive most often mentioned for continuing the practice of FGM was persuasion from older female relatives. Several women mentioned that their grandmothers played a significant role. One woman was subjected to FGM while living with her grandmother when her parents were migrating to Europe. The grandmother initiated the FGM without asking for the parents’ permission.

The mothers of the participants were described as having a main role but in an ambivalent position. Almost all the narratives included love and compassion for their own mothers when describing the decision process around FGM. The participants often reported that their mothers were under the power of their own mothers (the interviewees grandmothers). The peer pressure was mentioned as a strong argument, although the mothers were resisting. And if the mothers were present at the time of the mutilation, they could make sure that a less severe type was performed. It was also described by some that the mutilation was hastened due to forthcoming migration.

*“I was like three years old, and my mother wanted to speed up the process because we were leaving for Sweden. She didn’t want to have that taboo feeling. That she hadn’t done it even though we were going abroad. She told me that there was not much that was cut. Rather that they had sewed everything together. To make it look covered somehow… And then she also told me that if she had more knowledge, she would never have done this. And I think she has said it to me many times.”* (Deeqa).

One of the participants described how she herself put pressure on her mother due to her strong wish to undergo FGM. She remembered how she wished to belong to the group and to not feel ashamed about being different from her peers.

The participants remembered how their mothers let them become genitally mutilated without themselves actually wanting it but trusting that this was the best for their daughter. One of the mothers were described as clearly against the practice: she later even lectured to newly immigrated neighbours in Sweden about the negative health consequences of FGM.

*“My mother didn’t want me to be mutilated but everyone else did it and everyone knew each other. She felt the pressure… She didn’t want me to be bullied. So, she did it for my own sake… Somali culture is special. Sometimes you have to, even if you don’t want to. She did her best. I am not angry at her.”* (Fatima).

One of the participants said that her mother was in favor of the tradition and made sure that it was done without the father*’*s knowledge since he was against the tradition. Two of the participants described how their grandmothers initiated the procedure without the permission of the parents. The fathers of the women were either not present or against the tradition to mutilate girls.

*“Dad was against it but was not there to stop it. My dad, grandfather and all the men in my family were totally against it… My grandmother did it behind my grandfather’s back and my mother did it behind my father’s back.”* (Deeqa).

### Processing FGM through life

Most women mentioned FGM as something they had gone through earlier in life, however later reconciled with. It was described by some as being a part of their identity, however its significance decreased over time, as several other parts of their identities became more prominent.

*“A big part of my identity now is being a mother… It* (FGM) *used to be a part of my identity but now I don’t consider it being an important part.”* (Ayaan).

Responding to an open question on how they perceive themselves in relation to FGM, several expressed that they did not see themselves as victims.

*“I do not feel like a victim, however it’s of course a part of me. A part of my life. That’s who I am today. I would not replace it for anything else, because I do not have anything else to compare with… I feel like a strong person who has gone through this and today I feel good. I have my family, my children, and a fantastic sex life.” (*Deeqa).

Most of the participants did not feel anger towards their parents that had let them undergo FGM. However, some of them expressed frustration. One participant found out that she was mutilated during a gynecological examination and described how mad it made her and that she thereafter talked to her parents about it.

*“In the beginning I was very mad. Now I don’t think about it. Well, I think I actually do… It took me some time. I was very mad in the beginning, and I didn’t want to talk to them at all. But then I saw how bad it made my mom and dad feel… Okay I know, It’s not them, it’s the culture. So really, it’s not their fault, it’s the culture. This has been happening for the longest time.”* (Senait).

One participant described how she was informed about FGM from a newly arrived family from Somalia. Before this encounter she did not perceive she had much knowledge about the motives of FGM. But through this relationship she learnt more about the culture, language and concept of purity linked to FGM. However, she found the practice of FGM very problematic and couldn’t understand why the teenage girls in that family protected the culture. During the interview she recounted a conversation she and her girlfriends had with them:

*“We thought, you can’t do that to people, but they said ‘we are clean… you can stick a match in us’. I answered ‘what, that’s not normal!’ But they were really proud and thought that you aren’t a woman if you aren’t like that* (infibulated), *that you aren’t clean and that the man should open you up, you shouldn’t have the temptation.”* (Khadra).

All participants in this study expressed a negative attitude towards the tradition of FGM. Their negative attitude was due to the health risks, pain and the unnecessary and old-fashioned tradition of controlling girls’ bodies. There were different opinions whether FGM continues to be practised among others in the diaspora. Some found it possible that this might occur, especially during vacation trips to other countries, while most of the participants believed that the tradition of FGM was abandoned after migration. They speculated that increased knowledge about negative health consequences and misconceptions regarding the necessity to perform FGM, as well as fear of punishment, probably were reasons to abandon the tradition after migration.

*“Yes, but of course. No, but maybe, it depends on the parents. If they’re conservative you know, they might take the girl back and do it. But maybe that’s not happening here. Everyone is scared too.“* (Fatima).

### Living with lifelong health consequences

#### Effects on menstruation, urination and sexual intercourse

Half of the participating women did not experience their menstruation as challenging at all. For those who experienced menstrual pain, two described it as being severe, whereas the remaining two explained that the pain was relieved with a regular pain killer or spontaneously decreased with age.

“*I had menstrual cramps but it got better with ibuprofen…yes a lot of ibuprofen*“ (Fatima).

One woman associated the menstrual period with severe pain to the degree that she did not know how to handle it. She remembered how she fainted from pain when in school, however did not think that her menstrual pain was any worse than others.

*“Each time I got my period, I felt I was going to pass out. I was very pale and it was so damn painful…”* (Ayaan).

Some of the participating women described that urination was time consuming and involved different measures and adjustments to be able to urinate.

*“… very little came out. It was very difficult to pee so of course I realized that I was different.” (*Amal).

*“Previously it felt like I needed to put pressure (on the bladder) to pee faster.”* (Hiba).

Most of the participants with previous sexual experiences described painful intercourses when they started to practice it, however this often decreased with time and/or after surgical deinfibulation.

*“It felt tight, it was really painful.”* (Zahra).

*“It was so painful in the beginning… We didn’t have much knowledge about sex at all…”* (Khadra).

#### Deinfibulation as a positive turning point

Deinfibulation refers to the surgical procedure where the scar tissue in the seal covering the infibulated vulva is opened. Most women had the procedure done in Sweden, however one woman had it performed in England. For some of the participants the operation was postponed because of traumatic memories from the FGM. The deinfibulation was performed either with local anesthetics or full anesthesia. Most women did not experience any discomfort after the deinfibulation, however one mentioned soreness in the area, which was relieved with anesthetic gel. One woman described a strong emotional reaction of relief after the deinfibulation. Most of the participants had the deinfibulation performed independently of marriage, however a few went through the surgery during pregnancy when married. Only one woman mentioned that she actively waited until she was married due to traditional expectations.

*“I wanted to do it… but there are prejudices if you have done it. Maybe you are not a virgin anymore and stuff like that…I didn’t want to do the operation and then get shit for it later, for something I did not do… I wanted to wait until I was ready* (married). *So, it took another 4 years.”* (Hiba).

All women experienced the deinfibulation as a positive turning point. Deinfibulation made vaginal intercourse possible and painless. Positive changes were described as being able to pass urine without the procedure taking a very long time and “not having to press” anymore when urinating.

*“It was an aha-experience to be able to pee without it taking so long… The urine stream came differently.”* (Amal).

Also, the pain that some of the participants had lived with during the menstrual period disappeared after the deinfibulation. Some of the women expressed that they did not understand until after the deinfibulation that the suffering they had experienced previously during urination and menstruation was not normal and not necessary to live with.

*“Prior to my deinfibulation I always had very painful periods. I thought it was normal.”* (Ayaan).

*“I used to pee so slowly…It’s more free now!… Previously I had to wait and put my finger like this to wash myself… Now I don’t need to. It goes really fast. I don’t know why I waited so long.”* (Fatima).

One of the participants explained that she was happy with her deinfibulation since it released the pain during intercourse. But later in life, after childbirth, she felt that her genitals were different and embarrassing, unlike before childbirth.

*“You know when you are mutilated, everything is sort of even and pretty down there. An opening that is not too wide. Now after giving birth to my children they didn’t sew it back as before. Now it is more open. Now the urine tract and everything is visible as it should be. Then of course that suddenly feels strange to me… because this is not the way I used to look.”* (Deeqa).

#### Lifelong learning about sexual pleasure

When addressing sexual function and perceptions of sexuality during the interviews, we recognized that most of the participants had reflected upon this matter in relation to FGM and further had elaborated on different explanations for sexual dysfunction. Several women mentioned difficulties imagining how their sexual life would have been without the experience of FGM. The women’s sexual experiences differed. Some women had experience of long-time relationships, whereas others historically had several different sexual partners. One of the participants who recently got married explained that she yet had no experience of sexual intercourse or masturbation. Most of the women could reach orgasm although the issue of reaching orgasm was challenging for some of the women who described the process as very time consuming.

*“I can achieve orgasm, but not so often. I feel limited in what I can do… I know that I should practice stimulating myself, but I don’t feel comfortable yet.”* (Senait).

The reasons behind the sexual challenges described differed among women, some related to inexperience, some related to the mutilation and some related to the partner.

*“I think it depends on the man. The father of my child was really bad at sex. He was not sensitive at all or interested in my emotions or satisfaction.”* (Zahra).

It was also described that they needed to explore their bodies on their own to gradually develop skills to better enjoy their sexual life. FGM being the clear cause of challenges in sexual enjoyment was also stated in few cases.

*“Now I understand my body much better. Even if I don’t have a clitoris, I know that I can reach orgasm. But I needed to practice a lot.”* (Khadra).

One participant recounted psychological suffering due to alleged problems associated to FGM. The woman expressed how bad she felt when people talked about the problems she was expected to have due the FGM. Hearing about the negative health consequences mainly related to sexual enjoyment, but also to urination and menstrual periods was difficult to relate to as she had not experienced those herself. Later, when she started to have sex, she felt very insecure due to all the negative “talking”.

*“I always tried to object when others talked about mutilated girls, like ‘they don’t feel anything’ and ‘they are not feeling well and have lots of problems down there’. I used to say that I don’t have any problems and I feel just fine!”* (Deeqa).

*“You know, you have been hearing all the time ‘you should not be able to feel anything, you have no feelings, you might as well read a magazine’ (while having sex). So, this is what you hear, and then you believe it. Or I didn’t think it would affect me, but apparently it did. You see, I was affected by that in an unconscious way.”* (Deeqa).

Due to a good relationship with her partner and after having explored her sexuality open-mindedly, she managed to improve her sexual self-image as well as sexual function and now described her sex life as fantastic.

### Encounters with healthcare providers

#### Being acknowledged in the encounter with healthcare providers

All participants had previously seeked healthcare on several occasions for obstetric care and/or due to gynecological problems. Encounters with healthcare providers emerged as either positive or mixed with negative experiences. Several of the participants expressed their own experiences of trust and feeling safe and comfortable in the encounter with healthcare providers. Women expected the healthcare provider to address the subject of FGM and do it with a respectful and professional manner, because it was difficult for themselves to broach the subject. On the other hand, if being asked, they had concerns and certain expectations of how to be asked about it. They wished that the issue of FGM was raised in a sensitive way when relevant.

In positive encounters they highlighted being acknowledged, referring to being asked about FGM or informed in a neutral way that they had undergone FGM. The participants also appreciated being provided with information in a sensitive and compassionate way by a knowledgeable person. This was often described as a feeling of being educated. Further, being referred to psychological counseling was also appreciated. All the participants described the encounters at a specialist clinic eliciting feelings of trust and comfort and being educated. Further, it also emerged that it was appreciated when not referring to FGM if not relevant during the healthcare encounter.

*“She knew that just because I was circumcised it does not define my whole personality or who I am. So, she treated me like I was just any person.”* (Ayaan).

#### Feeling ignored

From the affected women’s perspective, not being asked or being asked about FGM status was a recurrent subject. On the one hand it was described that not being asked about it made them feel ignored. Feeling ignored was also experienced when healthcare providers during gynecological examination did not mention the fact that the woman had undergone FGM. Khadra, a woman with four children, had never been asked about FGM:

*“It feels like they don’t see you… It’s like, you are looking at my private parts…You are the one with more knowledge. It’s like not asking a woman with bruises if she has been abused!… I think it is inhumane because they could change someone’s life.”* (Khadra).

Experiences of feeling ignored were further expressed by several participants during gynecological examination and delivery. Some participants did not feel included in the reasoning about specific situations. One woman overheard conversations from the corridor about herself and how the caesarean section was decided on due to the FGM, something that was not explained to her. That feeling of being ignored was also experienced by another participant during delivery. She perceived that the staff did not explain why so many people examined her.

*“Doctors and midwives were running in and out (from the delivery room) and everybody said: We do not know how to fix this.”* (Ayaan).

A couple of the participants commented that they would have appreciated it if psychological counseling was being offered when seeking medical advice.

*“They just think ‘We are going to fix this person, just open her up and everything is over.’ But when they opened me floodgates of shit came out! My memories came back, that I thought I had forgotten.”* (Ayaan).

#### Experiences of insulting attitudes

Delayed care-seeking related to the FGM experience was expressed. Memories from the FGM event in childhood was explained as a reason to avoid seeking care for symptoms such as sexual dysfunction and menstrual pain. But seeking care was also avoided by some due to prior experiences of insulting attitudes. The silence from healthcare providers; not explaining, asking, or including the women in the decision making, was expressed as offensive by some of the participants. Furthermore, several participants experienced comments from healthcare providers that they perceived insulting.

*“I remember her comment… ‘This was tight!’ And I was like, ‘what is she saying?’… I felt so embarrassed, why did she say that? But I never understood that I was mutilated. She didn’t tell me. Maybe she didn’t understand that I was mutilated either… So, I thought this was normal… I felt uncomfortable, I never wanted to go to the gynecologist again.”* (Senait).

But for some it was also perceived as insulting when seeking health care for other reasons than FGM, but still offered care for the FGM on the initiative of the healthcare provider. For example, one woman booked an appointment due to symptoms of urinary tract infection, but was told about the advantages to reconstruct her clitoris:

“*The doctor talked a lot about my mutilation, that I could seek medical care. And they could help me get my clitoris back. And that they could help me look normal again… Sure, I was not angry with him, since I understood that he only wanted to help me. But I went there to talk about my urinary tract infection, not about my mutilation. If I needed antibiotics or something. Not to get help to look normal.* (Deeqa)

#### Feeling as of having no choice

One of the participants recalled that when she was a teenager, she had severe menstrual pain and was referred to a gynecologist by the school nurse. She said she was offered to have a deinfibulation operation performed, but the healthcare personnel did not understand the sensitivity of the cultural situation as her mother was present during the consultation.

“*She examined me and said ‘you have the choice if you want it or not.’ But my mother was with me, so I did not have much of a choice. This was before I got married.”* (Fatima).

## Discussion

There was a variety in experiences and perceived health consequences among the participants in our study, although the majority had undergone FGM type 3. The women expressed both positive and negative experiences of encounters with healthcare providers. They further described reflections and thoughts regarding the practice of FGM and their own experience in relation to everyday life. FGM was considered being a part of their life and identity, however with fading significance.

### Reflections on the tradition of FGM

In this study, FGM was mainly expressed as something the women had gone through in the past, and now reconciled with. FGM was further expressed by some as being part of their identity without being their only identity. Several participants talked about the practice of FGM as a social convention. It caused them both frustration, but also a way to understand why FGM continued to exist despite the society’s awareness of negative health consequences. All participants expressed negative attitude towards FGM.

Preserving virginity was described in this study as one of the main motives for performing FGM, which is in line with reports from the WHO [1]. In many communities, female virginity is considered an absolute prerequisite for marriage, and the family’s honor is dependent upon a girl’s virginity [33, 34]. Infibulation (FGM type 3) is associated with women’s virginity and virtue, but also men’s sexual pleasure [35–37]. An intact infibulation at marriage is proof of her virginity and high moral standards [38, 39]. According to the WHO, infibulation is considered the most severe type of FGM, mostly practiced in the north-eastern region of Africa; Djibouti, Eritrea, Ethiopia, Somalia and Sudan [1].

The participating women’s mothers’ ambivalence in the decision-making process regarding having their daughter undergo FGM or not, emerged in this study. Most of the participants explained the difficulties their own mothers had when deciding to have the procedure done, as the decision process to a great extent was governed by other female family members, social pressure, fear of harassment and exclusion. One of the participants expressed that fear of exclusion and the strong wish for her to belong, contributed to her mother’s decision of her undergoing FGM. It has previously been well described how FGM is motivated as a way to secure a good life for one’s daughters, despite having a negative attitude towards the practice [40]. Even post migration, social pressure to perform FGM on one’s daughters have been shown in several Nordic studies. The main risk has been described when revisiting the original country and being under the influence of relatives [34, 41].

The fathers of the participants on the other hand were either not present or strongly against the tradition. Men’s rising negative attitude towards the tradition has been shown in previous studies [42, 43]. A systemic review from 2015 found that education, knowledge of the health complications of FGM, age, religion, urban living, and migration influence men’s stated support for the abandonment of FGM [44].

### Health consequences

In the present study several negative lifelong health consequences were presented, all confirmed by previous literature [3–6]. Previous research has suggested that the complications are in relation to the severity of FGM [4, 8, 9]. In our study, the majority of participants were subjected to infibulation. However, some of them experienced long-term suffering whereas others perceived the consequences as minor. This variety of experiences in relation to FGM, despite having undergone the same type, is an important finding showing the heterogeneity between individuals. Another interesting finding in our study was that several participants expressed that they did not understand until after their deinfibulation, that previous symptoms regarding menstrual- and urinary problems was in fact associated with their FGM status. They might not have expressed problems regarding urination during the clinical consultation, yet experienced relief or positive changes after the surgery. On the other hand, some women might associate certain problems, such as menstrual pain, with having undergone FGM, although this relation is difficult to confirm medically. Altogether, these multidimensional and complex aspects of FGM contribute to uncertainty of the relation between FGM and perceived symptoms, as well as negation of symptoms.

An even more complex issue is sexual function in relation to FGM. Some women mentioned normal function and ability to feel pleasure and reach orgasm whereas others found it challenging. Several of the participants described that vaginal sexual intercourse was possible to perform without pain after deinfibulation, which had not been the case prior to surgery. However, a finding in our study was how other different aspects of sexuality, not directly related to FGM, was described as having impacted the woman’s sexual function. Factors mentioned included sexual self-image through life, inexperience of masturbation and sexual relations, as well as the relationship with their partner. One participant expressed that she had been influenced by negative expectations of presumed sexual dysfunction due to having undergone FGM, which affected her negatively in the beginning of her sexual career. She was frustrated by this since she later discovered that she after sexual self-exploration and with a good relationship actually did not experience any problems with her sexual life. This is an example of how expectations and views on sexuality in society also can play a role for a persons perceived sexual function.

### Medical deinfibulation

In this study, all women except one, had undergone infibulation and later in life a medical deinfibulation. Deinfibulation was perceived as a turning point in this study and all women expressed satisfaction with the result as it relieved symptoms and increased life quality. They described improvement regarding urination, menstruation and sexual intercourse.

Medical deinfibulation is a simple surgical procedure that can be performed on a woman previously subjected to infibulation. During deinfibulation, the covering seal consisting of the labia that were joined together during the mutilation, are surgically opened, in order to relieve passage of urine and menstrual blood and to enable vaginal intercourse and vaginal birth [45]. Medical deinfibulation should not be confused with the so-called traditional deinfibulation; when the partner is expected to widen the bride’s narrowed vagina through penile penetration. In this study traditional deinfibulation was never mentioned by the participants, whereas medical deinfibulation was seen as a positive option. This is in contrast with previous studies conducted in Sweden and Norway [35, 37]. In the Norwegian study it was shown that medical deinfibulation was considered a threat, undermining men’s attempt to prove their virility and manhood through the traditional penile penetration. Furthermore, it was described that the larger orifice created by a medical deinfibulation would result in a lesser tight opening, thus jeopardizing male pleasure [37]. In the Swedish study women’s perception of medicalised deinfibulation was strongly influenced by the importance of being a virgin and being able to prove their virginity [35]. Differences in views on medical deinfibulation between our studies could be related to time in the new country after immigration. Time is often discussed as a factor for changing attitudes regarding FGM [46–48]. Our study included participants that at the time of the interview had lived in Sweden between 10 and 32 years (average 24 years), while participants in the study by Chavez et al. [35] included women that had lived in Sweden between 9 months and 6 years (average 4 years). Another difference worth mentioning is that most of the participants in our study had lived in Sweden from a young age. In a study from UK exploring experiences and attitudes related to FGM in association with age at arrival in the new country, they concluded that living in UK from a younger age appeared to be associated with abandonment of FGM [49]. Living in a new country where FGM is perceived as something harmful could provide opportunity to reflect on one’s own experience of FGM as well as on traditional values. Medical deinfibulation independently from marriage might be interpreted as a step towards taking a stand against old traditions, thereby reclaiming the body and autonomy.

There was a variation between the narratives regarding views on the timing of medical deinfibulation. Despite a resistance to all forms of FGM, some found themselves in a limbo between traditional norms in the countries of origin and Swedish norms. While most women had the deinfibulation done independently of marriage, mainly due to physical problems, others waited until they were married. Out of them, only one woman explained that she actively avoided premarital deinfibulation, in order not to be accused of premarital sex. This is in contrast to other Scandinavian studies regarding premarital deinfibulation, where the women felt hesitant to undergo medical defibulation due to traditional perceptions and values of virginity [35, 37].

Although all women expressed satisfaction with having had a deinfibulation, one of them mentioned thoughts about her genital appearance later in life after childbirth, and a feeling of being too open and exposed. This is an issue that sometimes is discussed in relation to surgical deinfibulation. We believe that it is of importance to prepare the woman for changes that might appear after deinfibulation, including altered urinary beam or in some cases the sense of increased amount of visible vaginal discharge. When deinfibulation is performed in connection with childbirth, it is further important to inform her about expected changes that often can occur after vaginal delivery independent of this intervention, such as dryness and feeling wide. Nonetheless, a clinical examination to exclude an undiagnosed perineal tear or non-optimal repair could sometimes be appropriate in such cases, in accordance with regular postpartum care.

### Healthcare encounters

Although positive encounters with healthcare services were reported in our study, most participants also recounted negative experiences. The participants described being ignored, not included in the decision process and poor attitude as major reasons for having a negative experience with healthcare providers. Furthermore, they found poor knowledge among healthcare providers, or fear of ignorance, as a hindering factor for seeking healthcare. This is in accordance with other studies conducted in Europe, where challenges in encounters between healthcare providers and women with experience of FGM have been highlighted [21, 23, 26, 50].

From prior studies on healthcare providers’ perspective, Swedish midwives and obstetricians have expressed lack of knowledge, lack of guidelines and inconsistent practice in care of women with FGM as major factors resulting in less-than-optimal care [51–53]. They also found interaction with the women complex, due to language barriers, cultural differences and because of sensitivity of the issue of FGM. The question about how and when to talk about FGM often brings uncertainties for healthcare providers. Lack of knowledge about FGM among healthcare providers is well described in several publications from other high-income countries [21, 54–58].

In our study a recurrent subject regarding health care encounters was how the issue of FGM was addressed. The women generally expected that this subject would be brought up by the health care provider in a sensitive way. They further expressed feeling ignored when not being asked when relevant. This finding is consistent with findings in other studies [50, 59, 60]. In the study by Omron et al. the women emphasized the importance of knowledge of the cultural setting and asking questions in a sensitive matter when caring for women with FGM. The importance of verbal and non-verbal communication was also highlighted here, in accordance with findings expressed in our study [60]. Altogether, these results highlight the complex matter of broaching the subject. Apart from education about FGM and clinical guidelines, there is evidently an urge for self-reflection and discussion regarding the healthcare provider’s attitude and approach towards the issue. In order to gain relevant information the caregiver needs to create a safe setting allowing the patient to share her experiences and needs. This is a necessity for providing qualitative care and to decrease future negative health consequences after FGM and thus have clear implications for practice. During the interviews the participants spoke about FGM as something that had harmed them physically, psychologically or in both ways. However, it was also described how the significance of being affected by FGM decreased with time and reconciliation and now described their overall wellbeing as good. Acceptance could be understood as a coping strategy used to move on, and self-exploration as well as attained knowledge, a way to reach bodily understanding. Other authors have described possible coping strategies identified in studies on experiences of FGM affected women in the diaspora. Jacobsen et al. found that although women recounted pain and discomfort as adults, they did not give it power in everyday life [14]. Similar to our experience in the clinical practice and during conducted interviews, the authors further mentioned that women often used laughter when they shared stories that were painful or experiences of absurd encounters with healthcare providers and reflected upon this as also being a possible coping strategy.

Further, positive encounters with healthcare providers also seemed to play a significant role in perceiving good health. This was in part due to the opportunity of accessing medical care such as surgery, but also largely due to the experience of being professionally treated. Thus, an important finding is that healthcare providers approach is crucial in making positive changes in the lives of women subjected to FGM. The healthcare encounter can therefore be seen as a possibility to promote improved health in several ways. Basic education about FGM and its consequences to healthcare providers is a prerequisite for the ability to provide good care.

### Limitations

The results of this study reflect the perception from a limited number of women, and mainly women that originated from Somalia and had been residing in Sweden for a long time. Due to this, our results may not be generalizable to newly arrived immigrant populations in Sweden, or other FGM affected populations in a general Western context.

In interview studies there is risk for so called interviewer bias when respondents and interviewers interact as humans. This interaction can affect responses and validity [28]. In this case, the interviewer had years of experience from working in the specialized FGM clinic and thus was well accustomed to taking the medical history as well as listening to experiences told by FGM affected women. We believe that having heard a vast variation of narratives including possible perceived health consequences after FGM and further being well familiar with this tradition in different contexts, results in an in-depth knowledge of the issue which facilitates a neutral approach during the interview situation.

One can further argue that women visiting a specialized FGM clinic do so because of problems concerning FGM, which thereby could contribute to a negative bias in how the experience and perception of FGM will be described. Women with a less severe form of FGM may never seek healthcare based on the FGM status. Further, the criteria for participants to be able to speak Swedish, without the need of an interpreter, also implies a potential bias regarding attitudes and views on FGM, as this suggests longer residency in Sweden. However, the decision to exclude women that could not express themselves in Swedish was discussed thoroughly. When using an interpreter during interviews, parts of emotions and stories may be lost in translation. Participants may also fear that confidentiality may be compromised if a third party is listening. These reasons contributed to the decision not to use an interpreter.

Although we strived for heterogeneity among participants, we found that a majority of them were of reproductive age, originated from Somalia as well as had a similar level of education. Despite this and the fact that participants were recruited from the specialized FGM clinic, we noted a variety of experiences and perceptions of FGM among the women, including perceptions of long-term health consequences, which to us is an interesting finding.

The women in our study were generally highly educated, which might influence perceptions of FGM and negative attitudes towards the practice. Further, the participants similar sociocultural specifics may reflect their concordant ascribed meanings of FGM. In our study six out of eight participants originated from Somalia, one from a Somali region in Ethiopia. The eighth participant was born in Eritrea. Explanations regarding preservation of virginity and culture and their mothers’ ambivalence towards the tradition was similarly described by all women, regardless of sociocultural background. Whether the results had been different with a more heterogen group of participants in regard to sociocultural background is difficult to assess.

There are different strategies to enhance quality in qualitative research and they often address multiple criteria simultaneously. One aspect is the researcher’s experience in the field, which makes it more likely to gain rich, detailed information from the participants [61]. The researchers in this study have an in-depth knowledge of FGM. Reflexivity strategies involve attending systematically and continually to the context of knowledge constructions and particularly to the researcher’s potential effect on the collection, analysis and interpretation of data. Reflexivity involves awareness that the researchers bring to the inquiry a unique personal background and set of values that can affect the research process. So called investigator triangulation was performed in this study. It refers to the use of two or more researchers to make analysis and interpretations decisions. The premise is that investigators can reduce the risk of biased judgements and interpretations through collaboration [62].

## Conclusions

This study illustrates that FGM is a complex matter causing a variety in experiences and perceived health consequences among women affected. The study also indicates that clinical encounters have the potential to be improved through increased knowledge of FGM among health care providers. Recognizing the vast diversity among women affected, exerting a sensitive approach and individualizing healthcare could improve perceptions of healthcare encounters.

Further, the positive attitudes to medical deinfibulation, including those performed independent from marriage, might be seen as a positive step towards taking a stand against old harmful traditions and reclaiming the body and autonomy.

### Electronic supplementary material

Below is the link to the electronic supplementary material.

Supplementary Material 1

## Data Availability

The data are available from the corresponding author on reasonable request.

## Abbreviations

FGM: Female genital mutilation
WHO: World Health Organization
PTSD: Post-Traumatic Stress Disorder
