# Supplementary material for: “A part of my life”. A qualitative study about perceptions of female genital mutilation and experiences of healthcare among affected women residing in Sweden
Source: BMC Womens Health. 2024 May 22;24:304. doi: 10.1186/s12905-024-03149-1 (PMC11110410; doi:10.1186/s12905-024-03149-1)
Supplement: Supplementary file 1 — Supplementary Material 1 [file 12905_2024_3149_MOESM1_ESM.docx]

Interview guide

Part A: Demographics data

Age, place of birth, education, profession, religion, years of residency in Sweden, marital status, children.

Part B: Three broad themes with probing open question
As example: Could you develop….? Would you like to explain that a bit more…? Could you maybe provide an example…?

1. The experience of FGM.

2. Thoughts about possible self-lived health effects due to FGM.

3. Perceptions of encounters with Swedish health care providers.
